# Scaled-Up Production and Tableting of Grindable Electrospun Fibers Containing a Protein-Type Drug

**DOI:** 10.3390/pharmaceutics11070329

**Published:** 2019-07-11

**Authors:** Panna Vass, Edit Hirsch, Rita Kóczián, Balázs Démuth, Attila Farkas, Csaba Fehér, Edina Szabó, Áron Németh, Sune K. Andersen, Tamás Vigh, Geert Verreck, István Csontos, György Marosi, Zsombor K. Nagy

**Affiliations:** 1 Department of Organic Chemistry and Technology, Budapest University of Technology and Economics (BME), Műegyetem rakpart 3, H-1111 Budapest, Hungary; 2 Department of Applied Biotechnology and Food Science, Budapest University of Technology and Economics (BME), Műegyetem rakpart 3, H-1111 Budapest, Hungary; 3 Oral Solids Development, Janssen R&D, Turnhoutseweg 30, 2340 Beerse, Belgium

**Keywords:** electrospinning, scale-up, processability, biopharmaceuticals, oral dosage form, grinding

## Abstract

The aims of this work were to develop a processable, electrospun formulation of a model biopharmaceutical drug, β-galactosidase, and to demonstrate that higher production rates of biopharmaceutical-containing fibers can be achieved by using high-speed electrospinning compared to traditional electrospinning techniques. An aqueous solution of 7.6 *w*/*w*% polyvinyl alcohol, 0.6 *w*/*w*% polyethylene oxide, 9.9 *w*/*w*% mannitol, and 5.4 *w*/*w*% β-galactosidase was successfully electrospun with a 30 mL/h feeding rate, which is about 30 times higher than the feeding rate usually attained with single-needle electrospinning. According to X-ray diffraction measurements, polyvinyl alcohol, polyethylene oxide, and β-galactosidase were in an amorphous state in the fibers, whereas mannitol was crystalline (δ-polymorph). The presence of crystalline mannitol and the low water content enabled appropriate grinding of the fibrous sample without secondary drying. The ground powder was mixed with excipients commonly used during the preparation of pharmaceutical tablets and was successfully compressed into tablets. β-galactosidase remained stable during each of the processing steps (electrospinning, grinding, and tableting) and after one year of storage at room temperature in the tablets. The obtained results demonstrate that high-speed electrospinning is a viable alternative to traditional biopharmaceutical drying methods, especially for heat sensitive molecules, and tablet formulation is achievable from the electrospun material prepared this way.

## 1. Introduction

Biotechnology-based medicinal products have exhibited spectacular growth over the past decade and are presently one of the most rapidly expanding segments of the pharmaceutical industry [1]. A significant challenge is maintaining the activity of biopharmaceuticals, like proteins and other biologics, during storage, shipping, and upon administration. In liquid dosage forms, biopharmaceuticals often show instability due to being prone to physical and chemical degradation [2]. Therefore, retaining the initial activity of biopharmaceuticals during product development is a cornerstone in their commercialization. The elimination of water from the formulations not only improves the stability of the biopharmaceuticals, but has additional benefits, like reduced transportation costs and easier handling and storage [3]. However, biopharmaceuticals are usually very sensitive to water removal due to their structural complexity. This poses a great challenge to finding a cost-effective drying method that is capable of dehydrating the molecule without inducing degradation, and which can produce a powder suitable for oral downstream processing (e.g., tableting).

Currently, the most widely used drying technologies employed to obtain solid biopharmaceuticals are freeze drying and spray drying, despite their disadvantages. Besides being a highly energy- and time-intensive batch process, freeze drying exposes biopharmaceuticals to freezing stresses that can cause degradation. On the contrary, spray drying can be operated continuously and is more economical, but the high drying temperature applied during the process can induce inactivation of heat-sensitive biomolecules [4,5]. Electrospinning (ES) is a novel and efficient continuous drying technology providing rapid and gentle drying at an ambient temperature. ES is a fiber production method based on the effect of a high voltage on highly viscous polymer solutions. The technology generates a dried product by elongation (due to the electrostatic forces) of the liquid feed into ultra-fine (generally < 10 μm [6]) jets, resulting in a large surface area that enables near-instantaneous drying at room temperature. Over the past years, a large number of papers have been published about the application of electrospinning for the solid formulation of various biopharmaceuticals, such as enzymes, peptides, proteins (e.g., monoclonal antibodies), oligonucleotides, and probiotics [7,8,9,10,11,12,13,14,15], showing the high interest in the application of ES for biopharmaceuticals. In order for ES to be applied for industrial use, it is necessary to scale-up the technology to achieve adequate production rates and develop downstream processing steps, e.g., milling for conversion of the produced fibers into powders suitable for powder filling (oral capsules and parenteral applications) and tableting (oral dosage forms).

**Figure 1:**
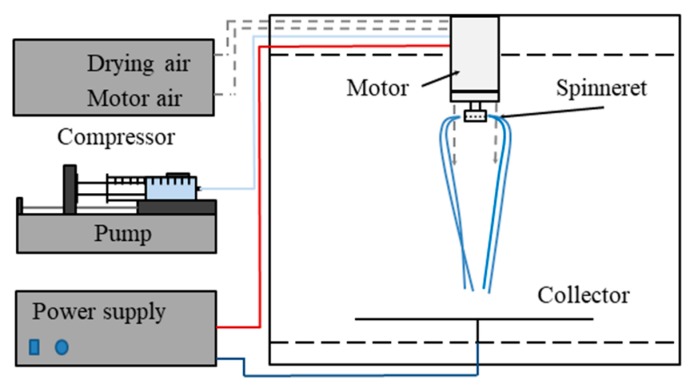
Schematic representation of high-speed electrospinning.

The laboratory-scale electrospinning device with a single needle has a rather low (0.01–2 g dry product per hour) productivity [14]. The scale-up of the technology is challenging, but a device has already been developed that uses high-speed electrospinning and is compatible with the requirements of the pharmaceutical industry [16]. With this method, productivity can be significantly increased by combining electrostatic [17] and high-speed rotational [18] jet generation and fiber elongation (Figure 1).

Another great challenge in addition to process scalability is achieving appropriate downstream processability of the electrospun fibers [19,20]. In this respect, the friability/grindability of the fibers and the properties (e.g., flowability) of the ground fibrous powder are also critical. A recent study by Hirsch et al. [21] evaluated the effect of various sugars and sugar alcohols on fiber friability in placebo fibers based on polyvinyl alcohol (PVA) and polyethylene oxide (PEO). They found mannitol to be the best friability increasing excipient due to its high crystallinity and the low moisture content in the fibrous samples. Mannitol-containing PVA- and PEO-based fibers were grindable directly after electrospinning, and there was no need for post-drying of the samples.

According to the authors’ best knowledge, there has been no attempt to develop grindable, and thus downstream processable, electrospun formulations of biopharmaceuticals. Therefore, the aim of this work was twofold: to develop grindable fibers containing a model biopharmaceutical produced by high-speed electrospinning and to produce a stable, solid oral formulation from the ground fibrous powder. Oral administration of biopharmaceuticals has many advantages, especially when targeted delivery to the colon is needed. The advantages include a high local concentration of the therapeutic agent, smaller dose, and reduced risk of drug interactions, besides limited or no systemic exposure to the biopharmaceutical, which is usually associated with toxicity and serious adverse effects, including immunogenetic responses and hypersensitivity reactions [11].

The model biopharmaceutical in the present work was a protein-type drug, β-galactosidase (lactase), which is an enzyme widely used as a drug for the treatment of lactose intolerance. It is estimated that about 70% of adults worldwide are not able to digest lactose due to the insufficient production of β-galactosidase in the colon, which brings on gastrointestinal symptoms when dairy products are consumed [22]. Structurally, β-galactosidase is a multidomain monomeric glycoprotein, which has been shown to inactivate during spray drying without excipients, due to surface denaturation [23]. Aggregation of the enzyme has also been observed during the storage of a freeze-dried formulation of β-galactosidase [24]. An earlier study demonstrated that the enzyme remained stable during electrospinning and storage [15].

## 2. Materials and Methods

### 2.1. Materials

Polyvinyl alcohol (PVA, M_w_: 130,000, 86.7–88.7 mol% hydrolysis) purchased from Sigma-Aldrich (Merck, Darmstadt, Germany) and polyethylene oxide (PEO, M_w_: 2 M) supplied by Colorcon (Dartford, UK) were used as polymer matrices. Mannitol (Mannogem EZ, SPI Pharma, Wilmington, DE, USA) was used as a grindable additive during electrospinning. Powder of β-galactosidase (opti-lactase A-100) from *Aspergillus oryzae* was kindly provided by Optiferm GmbH (Oy-Mittelberg, Germany; min. 100,000 FCC Unit/g). O-nitrophenyl-β-D-galactopyranoside (ONPG) was obtained from Carbosynth (Compton, UK). Microcrystalline cellulose (MCC) (Vivapur 200) was purchased from JRS Pharma (Rosenberg, Germany). Crospovidone was obtained from BASF (Ludwigshafen, Germany). Mannitol (Pearlitol 400DC) used as a tableting excipient was a kind gift from Roquette Pharma (Lestrem, France). The water used was from a Millipore Milli-Q ultrapure water system.

### 2.2. Scaled-Up Electrospinning of β-Galactosidase

The scaled-up electrospinning experiments were performed using a lab-scale high-speed electrostatic spinning (HSES) setup (Figure 1) consisting of a circular-shaped, stainless steel spinneret connected to a high-speed motor [16]. The rotational speed of the spinneret equipped with orifices (number of orifices: 8, diameter of the orifices: 330 μm, diameter of the spinneret 34 mm), combined with the effect of the electrical field, allowed increased productivity. PVA and PEO were added to purified water and the mixture was dissolved under heating (40 °C) and stirring (600 rpm). After complete dissolution, the solution was cooled down to room temperature and mannitol and β-galactosidase were added to the mixture, which was stirred (600 rpm) without heating until complete dissolution. The enzyme-containing polymer solution was fed with an SEP-10 S Plus syringe pump (Viltechmeda Ltd., Vilnius, Lithuania) with a 30 mL/h feeding rate. The rotational speed of the spinneret was fixed at 8000 rpm. The applied voltage was 37 kV during the experiments using a high-voltage power supply (Unitronik Ltd., Nagykanizsa, Hungary). A vertical drying air flow (2 bar) and the electrostatic forces directed the fibers to the grounded metal collector covered with aluminum foil, which was placed at a fixed distance (35 cm) from the spinneret. The experiments were performed at room temperature (25 °C).

### 2.3. Scanning Electron Microscopy

The morphology of the electrospun samples was studied by a JEOL 6380LVa-(JEOL, Tokyo, Japan) type scanning electron microscope in a high vacuum. Conductive double-sided carbon adhesive tape was used to fix the samples, which were subsequently sputtered by gold using ion sputtering (JEOL 1200, JEOL, Tokyo, Japan). A 15 kV accelerating voltage and 10 mm working distance were used during the measurements.

### 2.4. Determination of Residual Water Content

The residual water content of the samples was measured right after the electrospinning process using a Sartorius MA40 moisture balance (Göttingen, Germany). The residual water content was determined based on the moisture loss of approximately 0.1 g sample after 10 min at 105 °C.

### 2.5. Grinding/Milling of the Electrospun Material

To assess the friability of the produced fibers, the electrospun enzyme-containing material was pushed through a sieve with a 0.8 mm hole size to make it suitable for blending with excipients. This kind of milling is conceptually similar to oscillatory or conical milling (both methods produce powder by pushing the material through a sieve) with respect to the achieved powder properties.

### 2.6. Modulated Differential Scanning Calorimetry (DSC)

Modulated differential scanning calorimetry (DSC) measurements were carried out using a DSC3+ (Mettler Toledo AG, Switzerland) DSC machine in TOPEM^®^ mode (sample weight was 5–15 mg, pierced pan, nitrogen flush, 50 mL/min). The instrument applies stochastic temperature modulation superimposed on the underlying heating rate. A 1 °C/min overall heating rate and 1 °C pulse height (which means that the temperature was modulated by ±0.5 °C) were used during the measurements. The pulse width (the frequency of the modulation) was fluctuating randomly between 15 and 30 s. The temperature was increased from 0 °C to 200 °C.

### 2.7. X-ray Powder Diffraction (XRPD)

A PANalytical X’pert Pro MDP X-ray diffractometer (Almelo, The Netherlands) using Cu-Kα radiation (1.506 Å) and an Ni filter was used to study the X-ray powder diffraction patterns of the samples. The applied voltage and the current were 40 kV and 30 mA, respectively. The reference and the fibrous samples were analyzed between 2 θ angles of 4° and 42°, in reflection mode with a step size of 0.0167°.

### 2.8. FTIR Measurement

Fourier-transform infrared (FTIR) spectra were collected using a Bruker Tensor 37-type FTIR spectrometer equipped with a DTGS detector (Bruker Corporation, Billerica, MA, USA). The samples were ground with KBr and cold-pressed (200 bars) into discs. The measurement was carried out in transmission mode, at a scanning range of 400–4000 cm^−1^ with a resolution of 4 cm^−1^.

### 2.9. Raman Mapping

For Raman mapping, the ground fibrous sample was compressed slightly to gain a flat surface of the material. Spectrum collection was carried out using a Labram-type Raman instrument (Horiba Jobin–Yvon, Kyoto, Japan) coupled with an external 532 nm Nd:YAG laser source and Olympus BX-40 optical microscope. A 100× objective (laser spot size: ~2 μm) was employed in the high-resolution measurements. Raman photons were dispersed with a 950 groove/mm grating monochromator, directing them to the CCD detector. The spectral range of 390–1500 cm^−1^ with a 1 cm^−1^ resolution was measured. A 1 μm step size in both directions was used and the collected map consisted of 31 × 31 points. The spectrum acquisition length was 30 s and it was accumulated two times in each mapping point. The classical least squares (CLS) method using the spectra of the reference substances was applied to evaluate the data.

### 2.10. Tablet Preparation

Standard convex-shaped tablets were prepared from powder composed of the ground enzyme-loaded electrospun material mixed with different excipients (MCC, mannitol, crospovidone) on a CPR-6 eccentric tablet press (Dott Bonapace, Limbiate, Italy) equipped with 14 mm concave punches using manual powder filling.

### 2.11. Determination of Enzyme Activity

**Figure 2:**
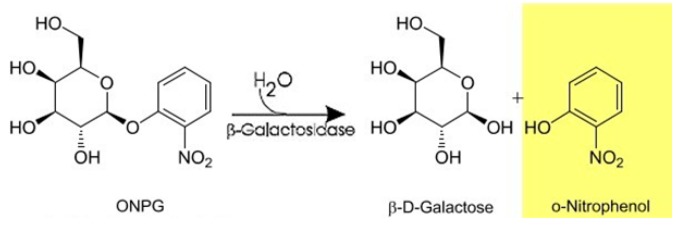
Enzymatic hydrolysis of O-nitrophenyl-β-D-galactopyranoside (ONPG) by β-galactosidase [20].

The activity of β-galactosidase was determined with ONPG as the substrate. ONPG is a colorless substance, which is cleaved by β-galactosidase to galactose (colorless) and o-nitrophenol (ONP, yellow, if pH ≥ 9) (Figure 2). The amount of ONP can be measured spectrophotometrically (at 420 nm, based on a previously created calibration (data not shown)). A total of 1.5 mL of a 6 M ONPG solution in 0.1 M acetate buffer (pH = 4.8) was pipetted to 0.1 mL of 10^−3^ g/L aqueous enzyme solution, preincubated to 55 °C. After running the reaction for 10 min at 55 °C, it was stopped by adding 1 mL of 1 M Na_2_CO_3_ solution to the reaction mixture. The solution was left to cool to room temperature and the absorbance of the sample was measured using a UV/V is spectrophotometer (Pharmacia Ultraspec III, Cambridge, UK). A stable enzyme formulation (over the course of the experiments) was measured in parallel to each fibrous enzyme-containing sample to serve as a reference. Experiments were conducted in quadruplicate.

### 2.12. Storage Stability Test

For storage stability testing, the reference enzyme formulation and the prepared enzyme-loaded tablets were kept in locked glass vials at 4 °C and 25 °C. The activity of β-galactosidase in the tablets was measured after 1, 3, 6, and 12 months of storage.

## 3. Results and Discussion

### 3.1. High-Speed Electrospinning of β-Galactosidase

The broadly applied single-needle electrospinning is not capable of the mass production of fibers, and therefore, its productivity is far from the needs of commercial pharmaceutical manufacturing. In this research, HSES was used to increase the throughput of the technology. Based on the results of our previous study [21] on placebo systems, a matrix solution composed of 7.65 *w*/*w*% PVA, 0.57 *w*/*w*% PEO, and 15.30 *w*/*w*% mannitol was selected for the experiments with β-galactosidase to achieve a grindable fibrous product.

The placebo system was supplemented with β-galactosidase powder so that the enzyme would be 20 *w*/*w*% of the solid product (Table 1).

Even though it was possible to obtain enzyme-containing fibers by electrospinning of this solution, the high solid content caused premature drying of the material, which resulted in blocking of the spinneret and it needed to be cleaned regularly during the electrospinning process. To address this problem, the amount of mannitol in the system was reduced so that, together with β-galactosidase, their amount would equal the amount of mannitol in the placebo system (Table 1). Electrospinning could be performed seamlessly using the optimized solution composition, which suggests that decreasing the amount of mannitol in the matrix leads to a better processability. However, in our earlier study, it was shown that when the sugar alcohol content in the fibers was decreased below a critical concentration, fiber grindability deteriorated [21]. Due to this, the mannitol amount was not decreased further in the present work.

**Figure 3:**
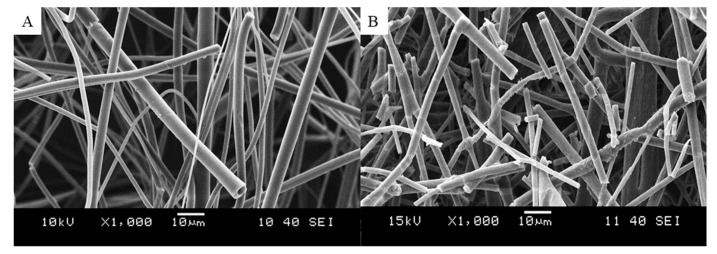
Scanning electron microscope images of β-galactosidase containing polyvinyl alcohol (PVA)-based fibers after electrospinning (**A**) and grinding (**B**) (at 1000-fold magnification).

The feeding rate used in the electrospinning experiment with the optimized composition was 30 mL/h, which is about 30 times higher than what is achievable with single-needle electrospinning for aqueous solutions [21]. The obtained fibrous mat was easily removable from the aluminum foil used on the collector (Figure S1). The product was examined by means of SEM. The fibrous nature of the produced β-galactosidase-containing sample can be seen in Figure 3A. Bead-free fibers were obtained with diameters around 1–5 µm, but submicronic fibers were also observable.

### 3.2. Processing of the β-Galactosidase-Containing Fibers

Processability of the formed fibers (e.g., milling, powder properties, etc.) is critical in the development of solid pharmaceutical products. The produced enzyme-containing fibers collected in the form of a fibrous mat were not suitable for conventional tablet production. Therefore, the collected mat needed to be ground to a powder before further processing. Grindability of the fibrous mats from the two matrix compositions containing β-galactosidase was evaluated right after electrospinning by pushing the material through a sieve with a pestle. The friability of the enzyme-containing fibers was sufficient without secondary drying, and the grinding of the mat resulted in a fibrous powder (Figure S1). It was noticed, however, that the fibers with the optimized matrix composition were slightly less friable than the fibers with the original composition. This suggests that less mannitol in the fibers results in decreased grindability, which is in line with our previous findings [21]. Further examinations were only carried out on the fibers with an optimized composition.

The morphology of the enzyme-loaded fibrous powder was studied with SEM (Figure 3B). It can be seen that the fibrous structure of the electrospun material was preserved during the grinding process and the diameter of the fibers was unchanged. However, grinding reduced the length of the fibers, resulting in a powder with improved flowability compared to the original unground material.

### 3.3. Characterization of the Fibers

**Figure 4:**
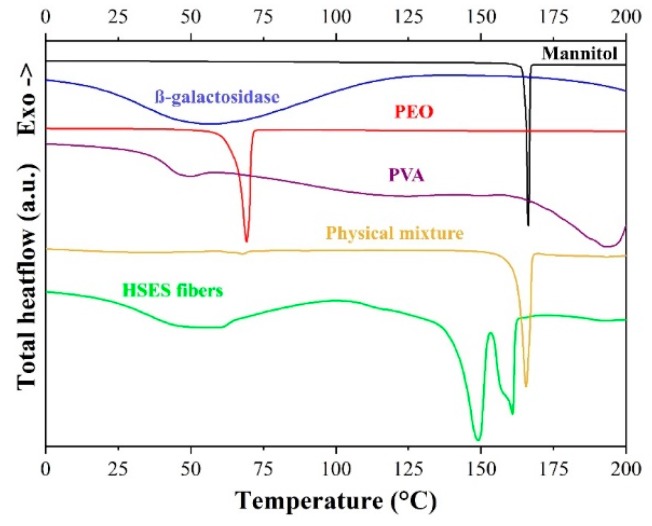
Differential scanning calorimetry (DSC) thermograms of δ-mannitol, β-galactosidase, polyethylene oxide (PEO), polyvinyl alcohol (PVA), the physical mixture of the fiber components, and the ground electrospun PVA + PEO + mannitol + β-galactosidase (high-speed electrostatic spinning (HSES) fibers).

In order to reveal the physical state of the different materials in the fibers, DSC, XRPD, and Raman examinations were carried out. The reference PEO and PVA are semi-crystalline polymers (glass transition temperature of PVA could be detected at 46.1 °C), which was confirmed by the DSC (Figure 4) measurement. The reference β-galactosidase powder did not show any significant peak (except for water loss). The reference δ-mannitol had a sharp melting peak at 165.9 °C, even though other researchers detected two peaks (the first belonging to the melting of the δ polymorph, followed by the fast recrystallization to the more stable β polymorph, with a second melting peak) [25,26]. Similarly, the fibrous material had two endothermic peaks at 148.8 °C and 160.8 °C, which probably belong to mannitol. During the DSC run, a melting point depression was seen (165.9 °C → 148.8 °C) due to the submicronic mannitol crystals with a large specific surface [27], which was probably followed by recrystallization to a more stable form and the melting of it [25,26]. Based on these results, it can be concluded that all fiber components are amorphous except mannitol.

**Figure 5:**
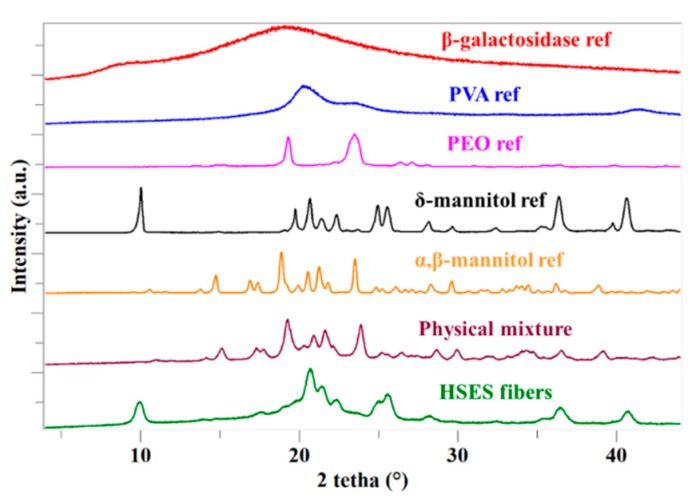
X-ray powder diffraction (XRPD) patterns of β-galactosidase; polyvinyl alcohol (PVA); polyethylene oxide (PEO); δ-mannitol; α, β-mannitol; the physical mixture of PVA, PEO, α, β-mannitol, and β-galactosidase; and the ground electrospun PVA + PEO + mannitol + β-galactosidase.

In order to confirm the results obtained by DSC, XRPD measurements were performed. According to the diffractograms, only mannitol was crystalline in the fibers, showing the characteristic peaks of the δ-polymorph. This polymorph of mannitol has been shown to be the least stable at ambient conditions [28] and it can transform into the α- or β-polymorph [26], which can be found in the physical mixture of the electrospinning matrix and β-galactosidase (Figure 5). During drying (e.g., spray drying), the formation of α- and β-mannitol is expected [29]. However, in the fibers, δ-mannitol can be found, which might be ascribed to the even faster drying with ES (and therefore, no possibility for rearrangement into a stable form) or to the presence of the other substances.

**Figure 6:**
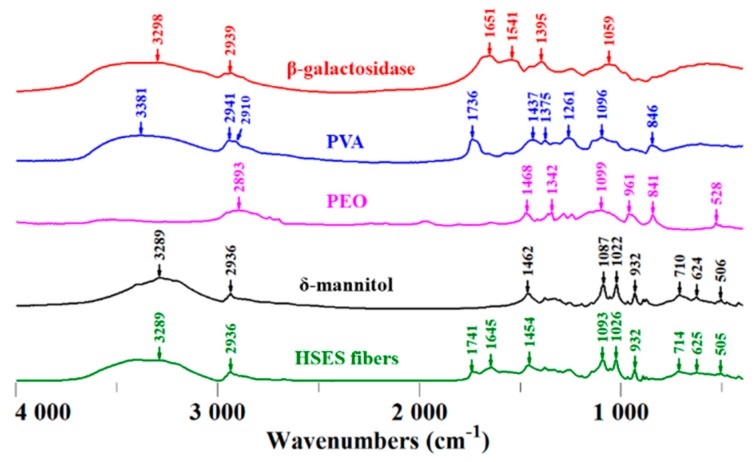
Fourier-transform infrared (FTIR) spectra of β-galactosidase, polyvinyl alcohol (PVA), polyethylene oxide (PEO), δ-mannitol, and the ground electrospun PVA + PEO + mannitol + β-galactosidase.

To evaluate the molecular interactions, FTIR spectroscopy was applied on the samples (Figure 6). PVA and mannitol molecules contain free hydroxyl groups (which can act as potential proton donors for hydrogen bonding) and β-galactosidase possesses numerous different groups that can act as potential proton donors or receptors. Therefore, hydrogen bonding might occur in the fibers. Characteristic absorption peaks of β-galactosidase are at 3298 cm^−1^ due to OH stretching and at 2939 cm^−1^ due to CH stretching. The absorption bands at 1651 cm^−1^ indicate the CONH vibration, and the 1541 cm^−1^ peak is the NH bending vibration of the β-galactosidase structure [30]. These peaks indicate the protein nature of β-galactosidase. PVA has a broad absorption band from OH at 3319 cm^−1^, bands from stretching vibrations of CH_2_/CH groups at 2941/2910 cm^−1^ and from C=O at 1736 cm^−1^ (characteristic of the carbonyl group of polyvinyl acetate), together with deformation bands of CH_2_/CH at 1437/1375 cm^−1^, and CO stretching vibrations at 1096 cm^−1^ and 1261 cm^−1^ [31]. Characteristic absorption bands of PEO include the band at 2893 cm^-1^ due to symmetric and antisymmetric CH stretching, and bands at 1468 cm^−1^ (asymmetric CH_2_ bending) and 846 cm^−1^ (CH_2_ rocking). The band in PEO at 1104 cm^−1^ indicates asymmetric COC stretching [32]. Mannitol showed the characteristic peaks of the OH group at 3289 cm^−1^ and the CH stretching at 2936 cm^−1^. Multiple characteristic absorption bands of δ-mannitol can be observed in the 500–1500 cm^−1^ region, which can also be seen in the spectrum of the electrospun sample, indicating the presence of the crystalline δ polymorph in the fibers [25]. The characteristic bands of β-galactosidase, PVA, and PEO either disappeared or appeared shifted in the spectrum of the HSES fibers, indicating molecular interaction (presumably hydrogen bonding) between the components.

**Figure 7:**
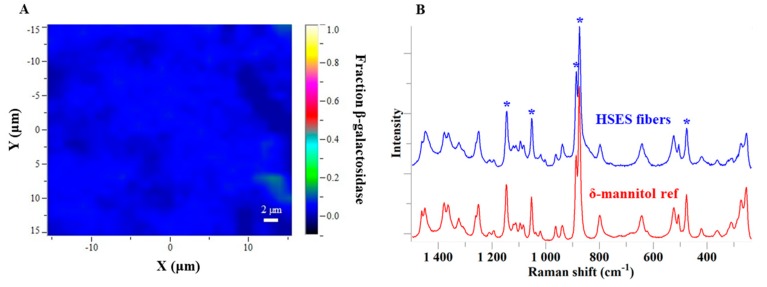
(**A**) Homogeneity study of β-galactosidase in the electrospun fibers by Raman mapping; (**B**) Raman spectra of the ground electrospun fibers and δ-mannitol (characteristic peaks marked with *).

The local distribution of the components in the ground fibers was analyzed by Raman mapping. For accurate dosing, homogeneity of the enzyme in the formulation is required. According to the Raman chemical map (Figure 7A), β-galactosidase seems to be uniformly distributed in the ground fibers as very small differences in color are seen.

The Raman mapping results also confirmed that the electrospun fibers mainly contained δ-mannitol. The characteristic peaks of the δ polymorph are shown in Figure 7B and these are in good agreement with data reported by others in the literature [33,34]. The ground electrospun fibers were reanalyzed by DSC, XRPD, and Raman after one year of storage at 4 °C. Even though the δ-polymorph is the least stable among the mannitol polymorphs, no recrystallization was observed in the fibers after this extended storage.

It has been previously shown that sugars and sugar alcohols can interact with water vapor and they have different water sorption capacities based on their physical state [35,36]. Amorphous sugars tend to absorb large amounts of water into their bulk structure, whereas crystalline sugars interact with water based on surface adsorption only. Water can act as a plasticizer in electrospun fibers and consequently, the water content of the electrospun materials influences their grindability significantly. It has been shown that a water content below 8% ensures acceptable grindability of sugar-containing fibers [21]. It was also shown that the physical state of excipients could impact the grindability, with crystalline mannitol eliminating the need for post-drying. The water content of the β-galactosidase-containing fibrous sample measured by the loss on drying (LOD) method was 6.0%. Presumably, this relatively low water content is due to the crystalline nature of mannitol in the fibers.

### 3.4. Tableting and Long-Term Stability Study of the Tablets

As the marketable final form of a lactase enzyme is preferably a tablet, the purpose of this study was not only to investigate the processability of enzyme-containing electrospun fibers, but also to produce tablets without losing the achieved advantages (i.e., activity preserved after processing). The fibrous powder was mixed with MCC, mannitol, and crospovidone, and the powder mixture was subsequently tableted (Figure S2). The main compression force was ~8 kN in this experiment. The composition of the produced tablets can be found in Table 2.

**Figure 8:**
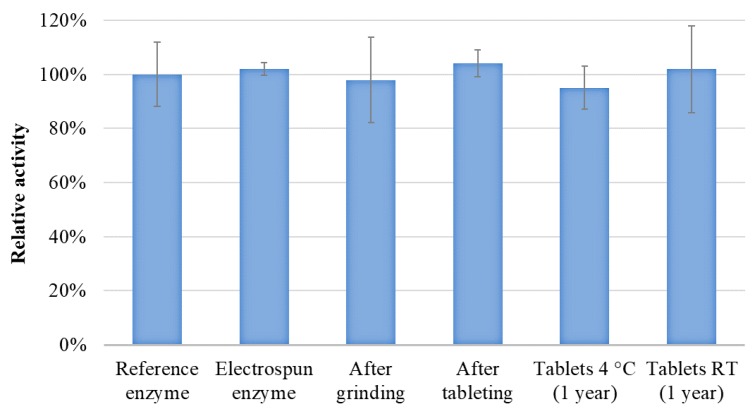
Enzyme activity of β-galactosidase in the fibers after high-speed electrostatic spinning (HSES), after grinding, after tableting, and the one-year stability result of the tablets (stored at 4 °C and room temperature).

Enzyme activity was measured after HSES, grinding, and tableting to assess the effect of the processing steps on β-galactosidase. The activity of a stable enzyme formulation was measured parallel to each sample to serve as a reference. The results are depicted in Figure 8. No significant difference can be seen between the activity of the reference enzyme and the electrospun and processed β-galactosidase, which suggests that the drying conditions with HSES are so gentle temperature wise that no degradation of this protein-type drug is seen.

Ensuring long-term stability of biopharmaceutical products is one of the main challenges in their pharmaceutical use and new formulations thus need to stabilize biopharmaceuticals to maintain their activity during storage. The storage stability of the electrospun and tableted β-galactosidase was compared with a reference enzyme formulation. The tablets of electrospun β-galactosidase were kept at 4 °C and room temperature and their activity were measured after 1 month, 3 months, 6 months, and 1 year. The periodic activity measurements showed that the enzyme remained stable in the tablets at both 4 °C and 25 °C, even after one year of storage (Figure 8). This result shows that the processable matrix containing PVA, PEO, and mannitol is suitable for stabilizing β-galactosidase in the long term.

## 4. Conclusions

The present work demonstrated that HSES is a feasible technology for producing biopharmaceutical-containing, processable fibers. A PVA-PEO-mannitol matrix was used to incorporate a model protein-type drug, β-galactosidase. A feeding rate of 30 mL/h was achieved in the experiments, which is 30 times higher than what is achievable for aqueous systems using single-needle ES. The produced fibrous mat was easily removed from the collector and it was found to be grindable without the need for a post-drying step, which simplifies downstream processing. All excipients were in an amorphous state in the fibers, except mannitol. The low water content and the crystalline mannitol in the fibrous sample could be the reason for the adequate grindability. The ground fibrous powder was mixed with tableting excipients and was successfully tableted. No decrease in enzyme activity was observed after either of the processing steps (electrospinning, grinding, and tableting). Besides, β-galactosidase remained stable in the tablets after one year of storage both at 4 °C and room temperature. In conclusion, the gentle drying by HSES and the processability of the applied matrix enabled the production of a final dosage form for the easy oral administration of this model protein without decreasing its activity.

## Figures and Tables

**Table 1:** Composition of the PVA-based electrospinning solutions of β-galactosidase.

Material	Amount (g)		Concentration (*w*/*w*%)		Ratio of Components in the Solid Product (%)	
	Original	Optimized	Original	Optimized	Original	Optimized
PVA 130,000	1.000	1.000	7.2	7.6	26.0	32.5
PEO 2M	0.075	0.075	0.5	0.6	2.0	2.4
Mannitol	2.000	1.300	14.4	9.9	52.0	42.3
β-galactosidase	0.770	0.700	5.6	5.4	20.0	22.8
Water	10.00	10.00	72.2	76.5	-	-

**Table 2:** Composition of the produced tablets.

Ingredients	Amount (mg)/Tablet	Amount (%)/Tablet
MCC 200	150	30
Mannitol	150	30
Crospovidone	50	10
Fibrous powder	150	30
∑	500	100

## Supplementary Materials

The following are available online at https://www.mdpi.com/1999-4923/11/7/329/s1, Figure S1. Electrospun sample removal from the collector and grinding process. Figure S2. Tablets prepared from ground fibrous enzyme-containing powder, MCC, mannitol, and crospovidone.
