# Supplementary material for: Scaled-Up Production and Tableting of Grindable Electrospun Fibers Containing a Protein-Type Drug
Source: Pharmaceutics. 2019 Jul 11;11(7):329. doi: 10.3390/pharmaceutics11070329 (PMC6680794; doi:10.3390/pharmaceutics11070329)
Supplement: Supplementary file 1 [file pharmaceutics-11-00329-s001.pdf]

pharmaceutics

MDPI

Supplementary Materials: Scaled-Up Production and Tableting of Grindable Electrospun Fibers Containing a Protein-Type Drug

Panna Vass, Edit Hirsch, Rita Kóczián, Balázs Démuth, Attila Farkas, Csaba Fehér, Edina Szabó, Áron Németh, Sune K. Andersen, Tamás Vigh, Geert Verreck, István Csontos, György Marosi and Zsombor K. Nagy

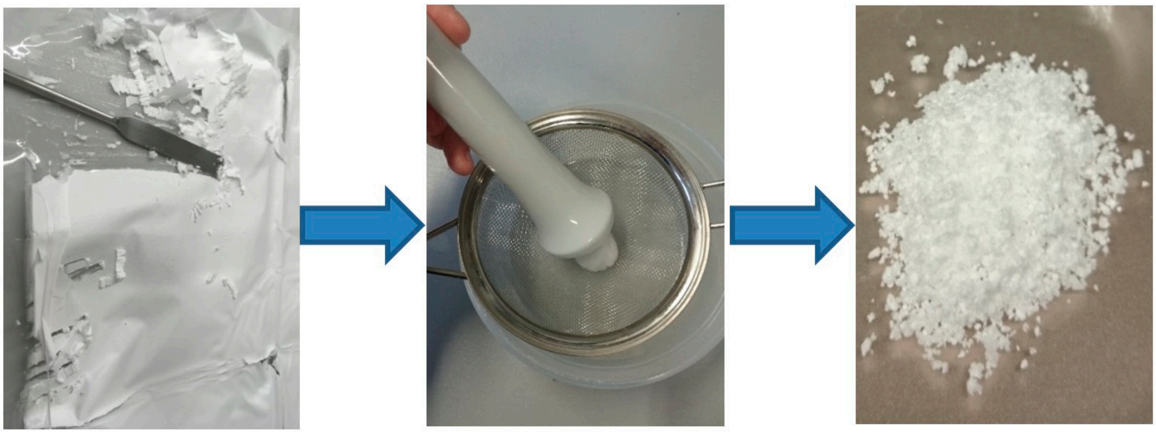

Figure S1. Electrospun sample removal from the collector and grinding process.

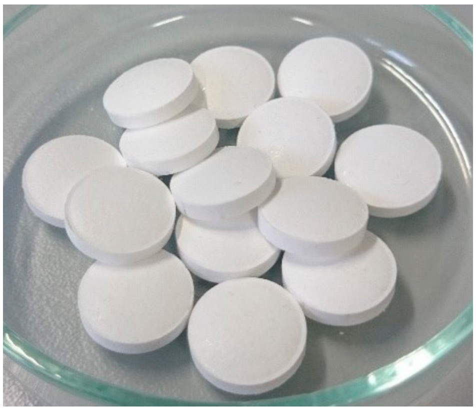

Figure S2. Tablets prepared from ground fibrous enzyme-containing powder, MCC, mannitol, and crospovidone.
